# Outcomes and management of delayed complication after severe blunt liver injury

**DOI:** 10.1186/s12893-022-01691-z

**Published:** 2022-06-22

**Authors:** Masaaki Kagoura, Kazuteru Monden, Hiroshi Sadamori, Masayoshi Hioki, Satoshi Ohno, Norihisa Takakura

**Affiliations:** Department of Surgery, Fukuyama City Hospital, 5-23-1 Zao, Fukuyama, Hiroshima 721-8511 Japan

**Keywords:** Bile duct injury, Bile leakage, Hepatic artery pseudoaneurysm, Liver trauma

## Abstract

**Background:**

The treatment of delayed complications after liver trauma such as bile leakage (BL) and hepatic artery pseudoaneurysms (HAPs) is difficult. The purpose of this study is to investigate the outcomes and management of post-traumatic BL and HAPs.

**Methods:**

We retrospectively evaluated patients diagnosed with blunt liver injury, graded by the American Association for the Surgery of Trauma Liver Injury Scale, who were admitted to our hospital between April 2010 and December 2019. Patient characteristics and treatments were analyzed.

**Results:**

A total of 176 patients with blunt liver injury were evaluated. Patients were diagnosed with grade I–II liver injury (n = 127) and with grade III-V injury (n = 49). BL was not observed in patients with grade I–II injury. Eight patients with grade III-V injury developed BL: surgical intervention was not needed for six patients with peripheral bile duct injury, but hepaticojejunostomy was needed for two patients with central bile duct injury. Out of 10 patients with HAPs, only three with grade I–II injury and one with grade III–V were treated conservatively; the rest six with grade III-V injury required transcatheter arterial embolization (TAE). All pseudoaneurysms disappeared.

**Conclusions:**

Severe blunt liver injury causing peripheral bile duct injury can be treated conservatively. In contrast, the central bile duct injury requires surgical treatment. HAPs with grade I–II injury might disappear spontaneously. HAPs with grade III–V injury should be considered TAE.

## Introduction

The liver is one of the most commonly injured organs in patients with blunt abdominal trauma [1]. Non-operative management is considered the first choice of treatment for hemodynamically stable patients with blunt liver injury [2]. However, in patients with severe blunt liver injury of grade III or greater (based on the American Association for the Surgery of Trauma (AAST) liver injury scale [3]), who do not respond to initial fluid resuscitation, an emergency operation should be performed to control bleeding.

Following blunt liver injury, bile leakage (BL), with an incidence rate that varies from 4 to 22%, is a major complication that leads to biloma and biliary fistula [4]. Although the clinical diagnosis of BL is difficult, a delayed diagnosis leads to high morbidity and prolonged hospital stay, making early diagnosis crucial for patients [5]. Previous studies have reported that bile duct injury following blunt liver injury may be managed by percutaneous drainage and endoscopic stent treatment [6–8]. Moreover, other aggressive treatments, such as partial liver resection, primary repair of the injured duct with T-tube insertion, and hepatectomy with hepaticojejunostomy, have also been reported to be successful in post-traumatic BL [9]. However, even if BL can be diagnosed during the initial operation, patients who have severe trauma are extremely unstable, which makes it difficult for surgeons to perform invasive surgery such as liver resection with reconstruction of the bile ducts.

Blunt liver injury results in intra- and/or extrahepatic bile duct injury, and the clinical management of an injured bile duct is controversial due to the variety of bile duct injuries. Few studies have evaluated the management of BL according to the location of the injured bile duct.

One of the other important complications after blunt liver trauma is hepatic artery pseudoaneurysm (HAP), which is rare but potentially fatal. Previous studies have reported that the incidence of HAPs after blunt liver trauma is 1–5% [10–12]. Symptoms of HAPs vary from asymptomatic to signs of rupture with intraperitoneal hemorrhage. Although symptomatic pseudoaneurysms should be treated, the indications for treating asymptomatic pseudoaneurysms are controversial because the natural history of pseudoaneurysms is still unclear. To our knowledge, few studies have examined the outcomes of HAPs after severe blunt liver injury [12].

To fill the gaps in knowledge, this study aimed to investigate the outcomes and management of BL and HAPs following severe blunt liver trauma.

## Methods

### Study patients and protocol

We retrospectively evaluated patients who underwent initial treatment for liver trauma in the Department of Surgery at Fukuyama City Hospital between April 2010 and December 2019. All medical records were carefully reviewed in this retrospective cohort study. We excluded patients with penetrating liver injury and those who died within 12 h of admission. Data from patients who were diagnosed with grade II or lower liver injury (grades I–II) and those who were diagnosed with grade III or higher liver injury (grades III-V) with severe blunt liver trauma were evaluated.

This study complied with the standards of the Declaration of Helsinki and the current ethical guidelines and was approved by the institutional review board of the Fukuyama City Hospital (approval #494). The requirement for informed consent was waived because this study did not report on a clinical trial and the data were retrospective in nature and analyzed anonymously.

### Classification of liver injury

**Fig. 1:**
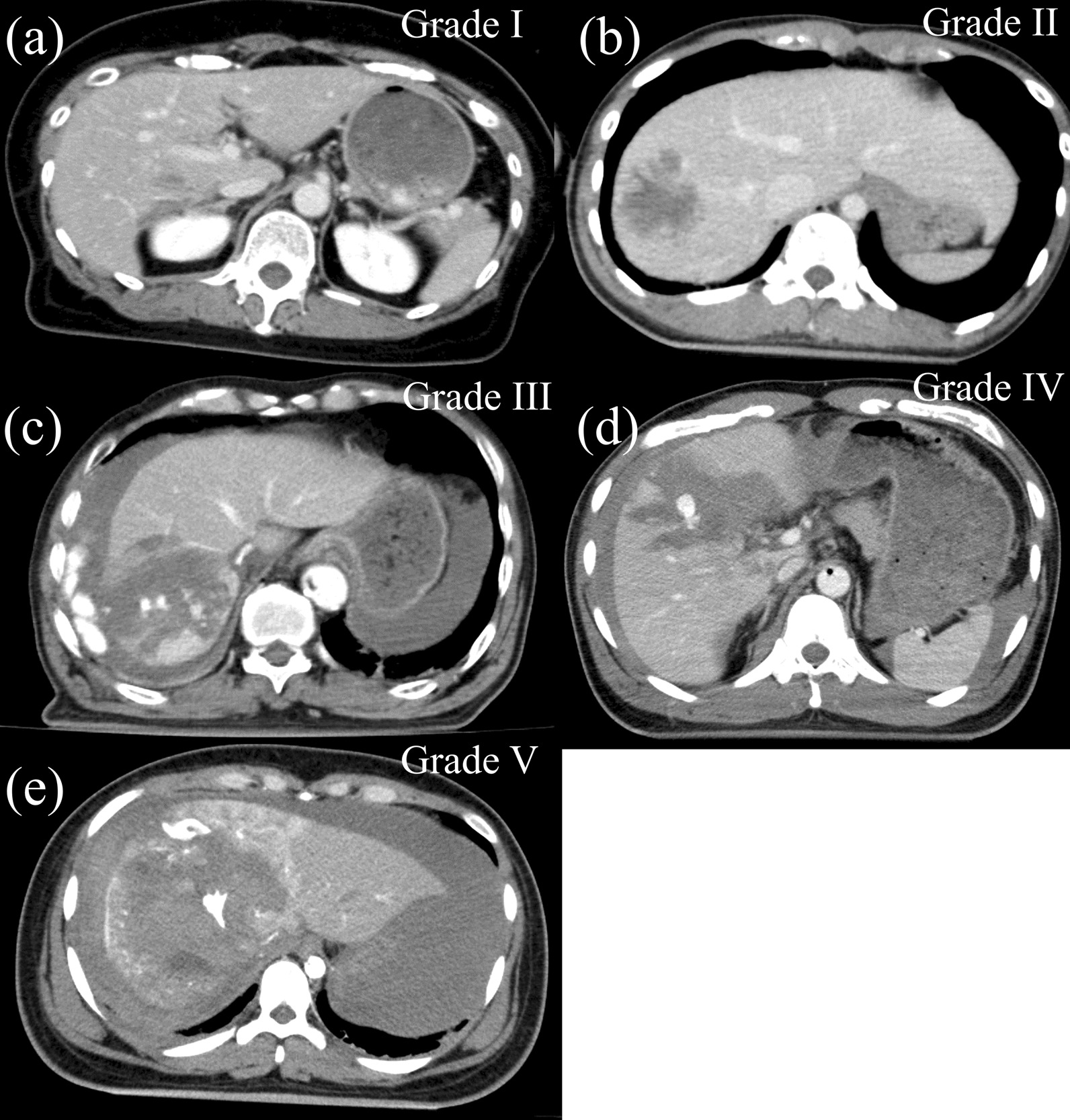
Different types of liver trauma. **a** < 1 cm depth laceration or < 10% surface area haematoma defines a grade I lesion. **b** 1–3 cm depth laceration or intraparenchymal haematoma defines a grade II lesion. **c** A laceration greater than 10 cm diameter or active bleeding within the parenchyma defines a grade III lesion. **d** Parenchymal disruption of 25–75% in a lobe defines a grade IV lesion. **e** Parenchymal disruption of greater than 75% in a lobe defines a grade V lesion

The grade of the liver injury was determined from a CT scan or laparotomy findings and based on the AAST liver injury scale. Liver injury was classified by a hepatic laceration to be superficial (lesser or equal to 3 cm in depth, grade I–II) or deep (greater than 3 cm in depth, grade III–V). A laceration greater than 10 cm diameter or active bleeding within the parenchyma defines a grade III lesion. Parenchymal disruption of 25-75% in a lobe defines a grade IV lesion. Parenchymal disruption of greater than 75% in a lobe defines a grade V lesion (Fig. 1). In Japan, patients with liver injury are usually classified according to the guidelines of the Japanese Association for the Surgery of Trauma (JAST); however, compared with JAST, the state of severe liver injury may be more clearly demonstrated by AAST; thus, we used the AAST liver injury scale in this study.

### Operative management

All patients with liver trauma were treated according to the hospital protocol based on the Japan Advanced Trauma Evaluation and Care program. The use of intra-aortic balloon occlusion (IABO) was determined by an emergency physician. Partial IABO was performed such that the balloon was occluded incompletely and inflated intermittently to avoid organ failure. When conditions permitted, nonoperative management was the primary treatment. Emergency laparotomy was performed in patients with hemodynamic instability and/or signs of peritonitis. Operative methods included perihepatic packing, hepatorrhaphy, liver resection, and hepaticojejunostomy. If we performed perihepatic packing as DCS to control hemorrhage, we planned to perform an operation for definitive treatment after 24–48 h and placed an intra-abdominal drain. If we performed a one-stage surgery, such as hepatorrhaphy, we placed an intra-abdominal drain.

### Bile leakage and hepatic artery pseudoaneurysm evaluation

The definition of BL after trauma is unclear in the literature; hence, the criteria of BL in this study were defined as follows: BL was defined as the presence of discharge through an intra-abdominal fluid drain, which has a bilirubin concentration at least three times higher than the serum bilirubin concentration, as the presence of fever and/or abdominal pain accompanied by intra-abdominal fluid accumulation detected by CT, or as cases requiring radiologic intervention or relaparotomy due to bile collection or bile peritonitis.

HAPs were detected using contrast-enhanced CT. We defined an aneurysm as localized widening of an artery or protrusion, either circumferentially or locally. Treatment was considered for all symptomatic HAPs if present, or if the diameter of the HAP was > 10 mm, transcatheter arterial embolization (TAE) was considered. Even small HAPs protruding into the damaged liver parenchyma were treated because of the risk of rupture due to bile exposure and infection. In addition, hemostasis due to increased internal pressure cannot be expected due to bleeding into the peritoneal cavity, in contrast to an intrahepatic parenchymal rupture. However, the treatment of HAPs depends on the patient’s condition or the location of HAPs.

Our CT follow-up timing was as follows: when we treated trauma patients conservatively (i.e., non-operatively), we performed the CT scans at least twice, namely on the day after the injury and again before discharge. In contrast, when patients were treated operatively, postoperative CT scans were performed based on clinical and/or laboratory suspicion of biliary complications and HAPs, including elevated temperature, abdominal pain, jaundice, or hemorrhagic shock. In fact, liver injury patients may have had multiple traumas, such as cerebral contusions, multiple bone fractures, and pelvic fractures in severe liver injury; these patients must be followed up frequently in collaboration with other departments.

### Data collection

**Fig. 2:**
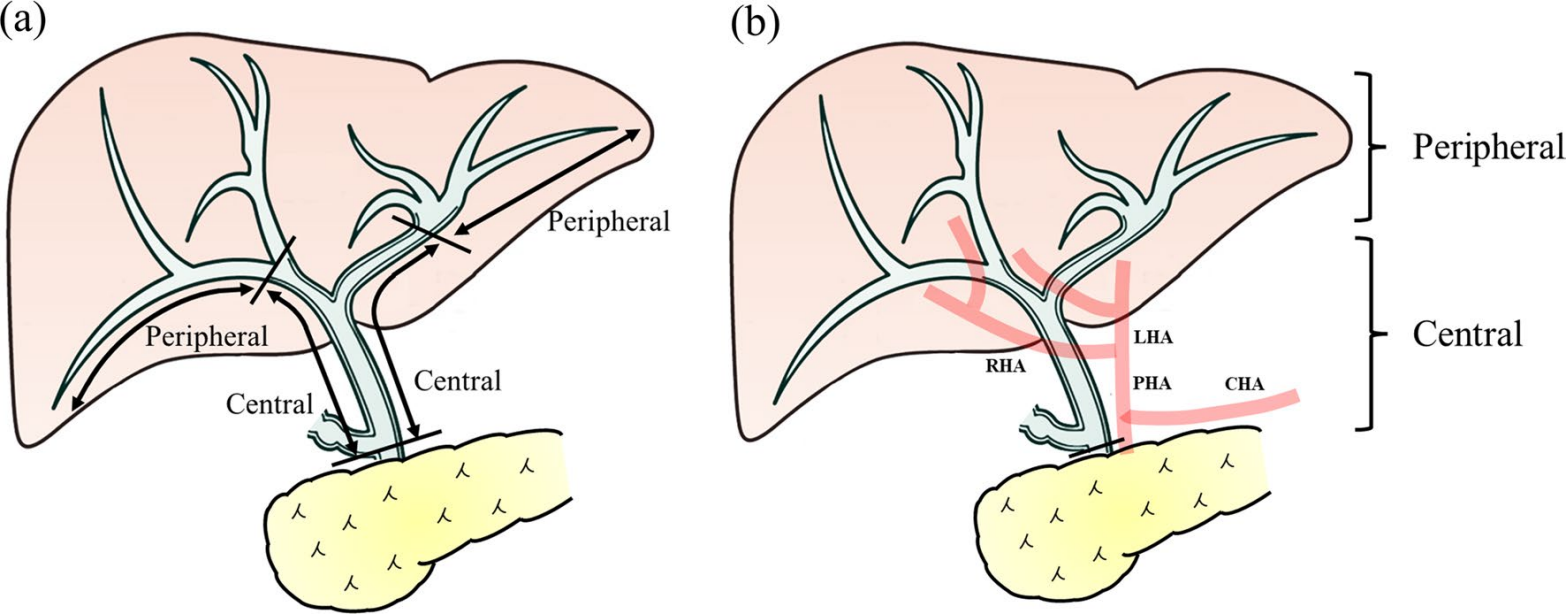
The definition of injured hepatic duct and location of hepatic artery pseudoaneurysm. **a** The central bile duct was defined as the region between the secondary branches of the right and left hepatic ducts and the common hepatic duct at the upper edge of the pancreas. The peripheral bile duct was defined as the region proximal to the central one. **b** The central hepatic artery was defined as comprising the common hepatic artery, the proper hepatic artery, and the left, middle, right, right anterior, and right posterior hepatic arteries. The peripheral hepatic artery was defined as being proximal to the central artery

We collected the following variables for analysis: age, sex, trauma mechanisms, injury severity score (ISS), presence/absence of an IABO, use of TAE, grade of liver injury, length of intensive care unit stay, length of hospital stay, mortality, and the type of treatment for BL. We further divided the cases of liver injuries into either peripheral or central bile duct injuries, determined through CT or laparotomy findings. The injured hepatic duct was defined as follows (Fig. 2a): the central bile duct was defined as the region between the secondary branches of the right and left hepatic ducts and the common hepatic duct at the upper edge of the pancreas, and the peripheral bile duct was defined as the region proximal to the central duct.

Concerning HAP, we also collected the following clinical factors: location of the pseudoaneurysm, pseudoaneurysm size, the number of pseudoaneurysm formations, and the type of treatment for HAPs. The definition of the location of the hepatic artery pseudoaneurysm was as follows (Fig. 2b): the central hepatic artery was defined as comprising the common hepatic artery, the proper hepatic artery, and the left, middle, right, right anterior, and right posterior hepatic arteries, and the peripheral hepatic artery was defined as proximal to the central artery.

Statistical analyses were performed using the SPSS version 26 software (SPSS, Chicago, IL). Comparisons between the two groups were conducted using Mann-Whitney U-tests. Quantitative values were expressed as median and range, as appropriate. Group differences in categorical outcomes were assessed using the chi-square test or Fisher’s exact test. The level of statistical significance was set at 0.05.

## Results

### Patient characteristics

The potential participants of this study included 190 patients. We excluded 9 patients with penetrating liver injury and 5 patients who died within 12 h of admission.

**Fig. 3:**
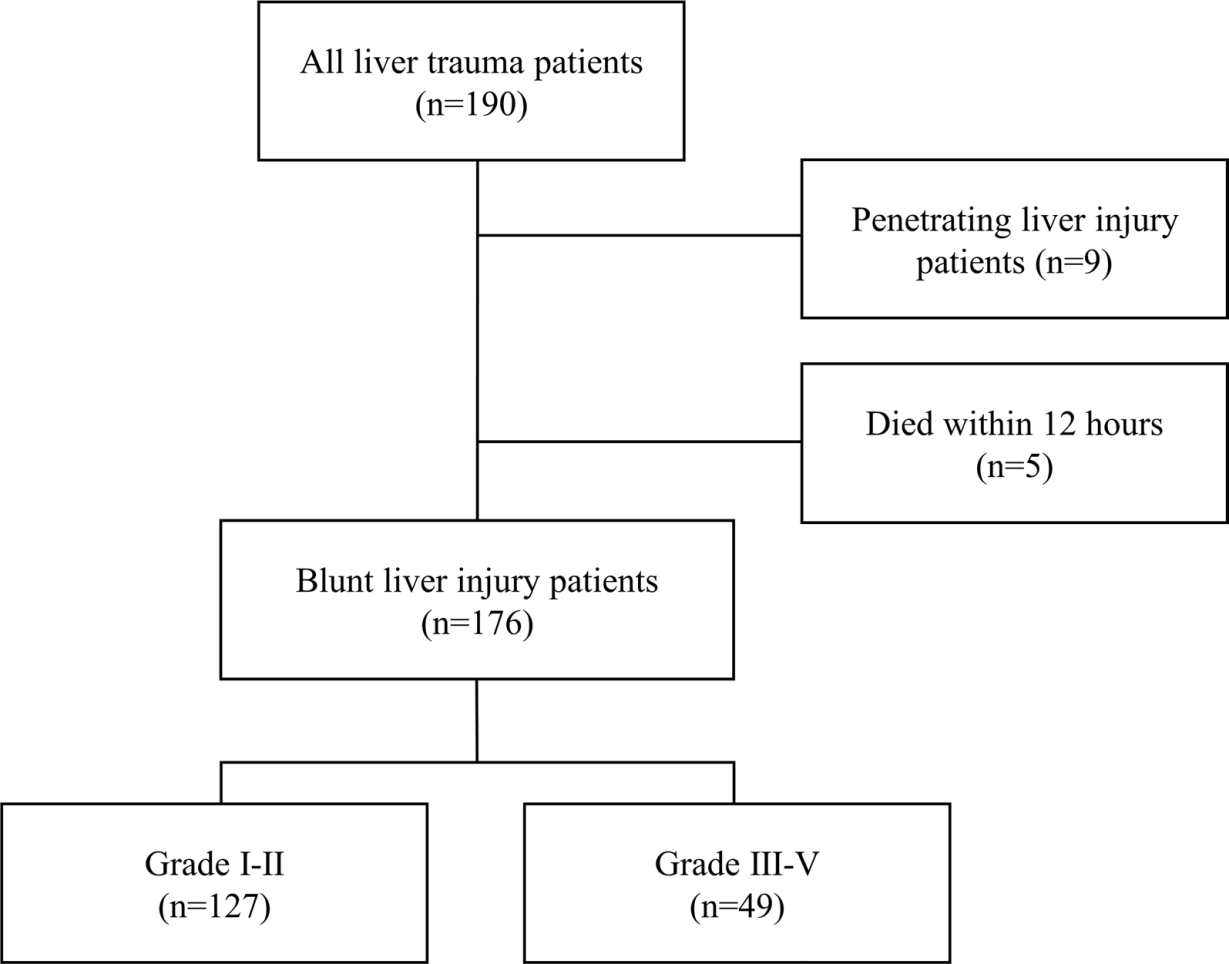
Flow chart of liver trauma patients in the study

The data evaluated from 127 patients with liver injury of grade II or lower (grades I–II) and 49 patients with liver injury of grade III or higher (grades III–V) and severe blunt liver trauma are shown in Fig. 3. The baseline characteristics of patients with blunt liver trauma are shown in Table 1. The proportion of patients requiring IABO (p < 0.001), TAE (p < 0.001), and operative management (p < 0.001) was significantly greater in the grade III-V injury group than in the grade I-II injury group. No significant difference in ISS was seen between the two groups. Of the 49 patients with grade III-V injuries, eight developed BL, but none of the 127 patients with grade I–II injuries developed BL. HAP appeared in three patients with grade I–II injuries and in seven with grade III–V injuries. BL and HAP were both significantly greater in the grade III–V injury group than in grade I-II (p < 0.001, p = 0.005, respectively).

**Table 1:** Baseline characteristics of patients with liver trauma

Patient number	Grades I–II (n = 127)		Grades III–V (n = 49)		P value
Age, years	36	[5–85]	46	[9–93]	0.092
Male sex	69	(54.3)	31	(63.2)	0.210
Trauma mechanisms					0.235
Traffic injury	88	(69.3)	36	(73.5)	
Falls	16	(14.2)	6	(12.2)	
Abdominal compression	4	(3.5)	4	(8.2)	
Other	19	(15.9)	3	(6.1)	
ISS	21	[4–75]	25	[9–75]	0.176
IABO	8	(6.3)	13	(26.5)	< 0.001
TAE	1	(0.8)	19	(38.8)	< 0.001
Liver injury grade					N/A
I	42	(33.1)	0		
II	85	(66.9)	0		
III	0		34	(69.4)	
IV	0		11	(22.4)	
V	0		4	(8.2)	
Initial treatment					< 0.001
Operative management	13	(10.2)	23	(46.9)	
Nonoperative management	114	(89.8)	26	(53.0)	
Result of injury					
Bile leakage	0	(0)	8	(16.3)	< 0.001
Hepatic artery pseudoaneurysm	3	(2.4)	7	(14.3)	0.005

Data are presented as the number of patients (%) or median value of the parameter (range). ISS: Injury Severity Score; IABO: intra-aortic balloon occlusion; TAE: transcatheter arterial embolization

### Outcomes of bile leakage

**Fig. 4:**
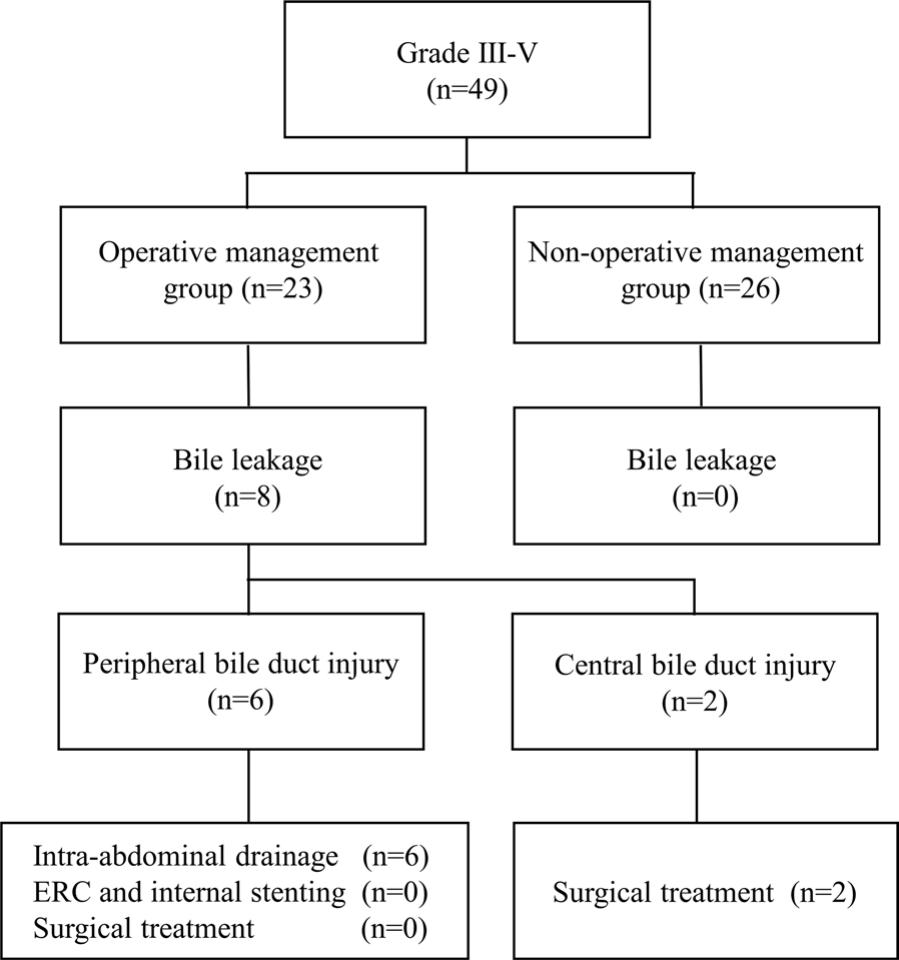
Outcomes and management of patients with bile leakage

The outcomes of BL in patients with grade III-V injuries are summarized in Fig. 4; Table 2. Eight patients (34.8%) with operative management developed post-traumatic BL, with the site of bile duct injury being the peripheral ducts in six patients and central duct in two patients. None of the patients with non-operative management developed BL requiring treatment. BL was diagnosed based on either intraoperative findings or a continuous biliary leak from an intra-abdominal drain in all but one of the patients, which was based on computed tomography (CT) scan findings (Table 2).

**Table 2:** Outcomes of bile leakage following blunt liver injury with a severity grade of grade III or greater

Bile leakage	n = 8	
Liver injury grade		
III	3	(37.5)
IV	4	(50.0)
V	1	(12.5)
Initial treatment for blunt liver injury		
DCS	5	(62.5)
Hepatorraphy	2	(25.0)
Cauterization	1	(12.5)
Diagnostic procedures		
Intraoperative findings	4	(50.0)
Biliary leak from the intra-abdominal drain	3	(37.5)
CT scan	1	(12.5)
Injury location		
Peripheral bile duct	6	(75.0)
Central bile duct	2	(25.0)
ISS	25	[10–75]
Duration of abdominal drainage, days	14	[11–42]
Length of stay in ICU, days	8	[2–28]
Length of hospital stay, days	40	[18–131]
Peripheral bile duct	28	[18–79]
Central bile duct	90	[49–131]
Mortality	0	(0)

Data are presented as the number of patients (%) or median value of the parameter (range). DCS, damage control surgery; CT, computed tomography; ISS, injury severity score; ICU, intensive care unit

### Management of bile leakage

BL was treated differently depending on the location of the bile duct injury (Fig. 4). In all patients with a peripheral bile duct injury, BL was managed without surgical intervention. In contrast, in two patients with a central bile duct injury, BL was controlled by hepaticojejunostomy or hepaticojejunostomy with liver resection. Of the two central bile duct injuries, in one patient with bilateral hepatic ducts and the common hepatic duct were torn separately, simultaneous reconstruction of the bilateral hepatic ducts was impossible because the condition was not permitted. Each stump of the injured hepatic ducts was closed using running sutures, and external biliary drainage for the bilateral lobes was performed on postoperative day (POD)2. After the condition was stable, we performed hepaticojejunostomy on POD89. In the other patient with the right hepatic ducts and the common hepatic duct were completely torn, biliary reconstruction in a one-stage surgery was performed because the patient’s condition was stable. None of the patients underwent endoscopic retrograde cholangiography (ERC) and internal stenting. No death was noted in patients with BL.

### Outcomes of hepatic artery pseudoaneurysm

**Fig. 5:**
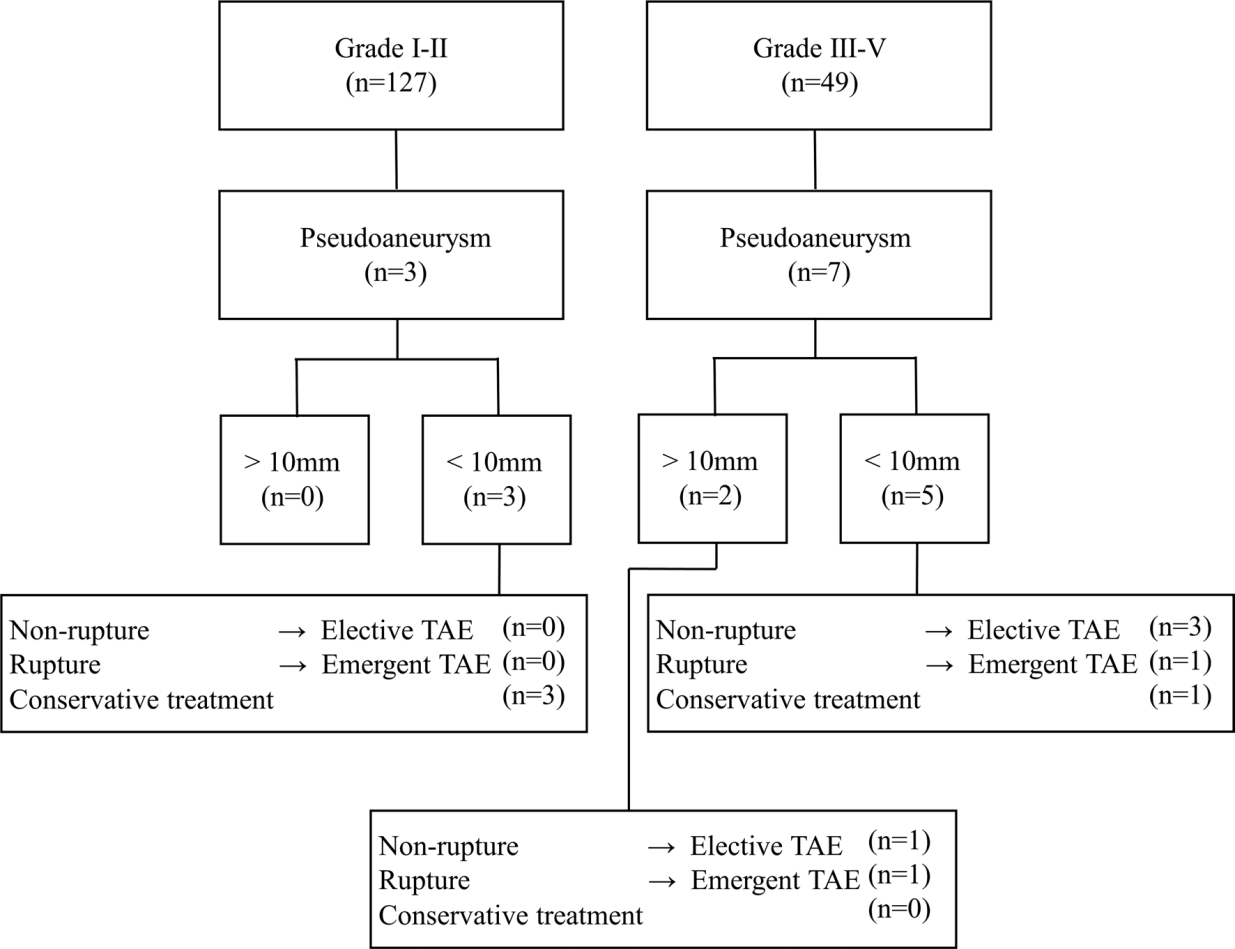
Outcomes and management of patients with hepatic artery pseudoaneurysm

The outcomes of the HAPs are summarized in Fig. 5; Table 3. All ten patients with grade I–V injuries developed peripheral HAPs. The median diameter of the aneurysm was 4.5 mm in the grade I–II group and 6.5 mm in the grade III–V group. Single pseudoaneurysms were detected in the grade I–II group, but multiple formations were observed in most cases in the grade III-V group.

**Table 3:** Outcomes of hepatic artery pseudoaneurysm following blunt liver injury

Hepatic artery pseudoaneurysm	Grades I–II (n = 3)		Grades III–V (n = 7)	
Liver injury grade				
I	0	(0)	0	(0)
II	3	(100)	0	(0)
III	0	(0)	5	(71.4)
IV	0	(0)	2	(28.6)
V	0	(0)	0	(0)
Initial treatment for blunt liver injury				
DCS	0	(0)	1	(14.2)
Cauterization	0	(0)	2	(28.6)
Non-operative	3	(100)	4	(57.1)
Location				
Peripheral	3	(100)	7	(100)
Central	0	(0)	0	(0)
Day of detection	6.5	[5–8]	8	[1–19]
Number of pseudoaneurysm formation	1		3	[1–6]
Diameter, mm	4.5	[3–6]	6.5	[3–15]
Mortality	0	(0)	0	(0)

Data are presented as the number of patients (%) or median value of the parameter (range). DCS: damage-control surgery

### Management of hepatic artery pseudoaneurysm

The management of the HAPs is summarized in Fig. 5. In the three patients with grade I–II injuries, all patients were treated conservatively, because the diameter of the HAP was < 10 mm and the HAP was located in the intrahepatic parenchyma. In the patients with grade III–V injuries, TAE was performed in six patients; in four patients, pseudoaneurysms were seen on the damaged edge of the liver with a risk of spontaneous rupture, and the other two patients underwent emergent TAE because of rupture. One patient with grade III-V injury was treated conservatively because performing TAE had a risk of liver necrosis; we identified portal vein thrombosis in the arterial territories where HAP was located, which may have led to hepatic necrosis. All TAE procedures for HAPs used coils and/or n-butyl-2-cyanoacrylate (NBCA) as permanent embolization, except for the one patient who could not undergo superselective TAE. Recanalization and rebleeding episodes did not occur in any of these cases. All pseudoaneurysms disappeared, and no recurrence was observed after discharge from the hospital.

## Discussion

In the current study, we demonstrated that, following an emergent operation for grade III or greater liver injury due to blunt liver trauma, a peripheral bile duct injury could be treated conservatively with intra-abdominal drainage. Alternatively, a central bile duct injury requires aggressive surgical treatment, such as liver resection with reconstruction of the injured bile ducts. To manage HAPs, we successfully treated all patients with or without TAE.

The liver is the third most commonly injured organ due to abdominal trauma, and almost all liver injuries are blunt injuries [1]. Severe blunt liver injury carries a high risk of mortality and often requires emergency surgery. The most important purpose of an emergent laparotomy is to rapidly control the bleeding through damage control surgery (DCS), but the injured bile duct is left untreated if the patient is not stable. Biliary peritonitis, intractable BL, and septic shock, which leads to high mortality rate, can occur following BL [13, 14]. HAPs also should be considered a fatal, delayed complication after liver trauma. HAPs sometimes present no symptoms until it has ruptured, and can lead to fatal outcomes owing to sudden severe hemorrhage. Based on these reported outcomes, it is evident that attention must be paid to the management of BL and HAPs following liver trauma.

In the current study, all BL with peripheral bile duct injury could be managed without additional surgical procedures. Previous studies have reported BL, even in sever liver injury, can be managed by conservative treatment such as ERC and internal stenting [6–8, 15, 16], however the appropriate timing for and the choice of ERC have not been established and are controversial. Patients with severe liver trauma sometimes have severe traumatic injuries to other organs, such as the brain or multiple bone fractures. In the current study, the median ISS in the grade III-V group was 25, which was relatively high. Thomas et al. reported that the mortality rate of patients with ISS > 16 was 20% [17]. Thus, the use of ERC and internal stenting is difficult in some patients with unstable conditions due to these injuries [18]. Moreover, one of the most fatal complications of ERC is ascending biliary cholangitis, which may lead to hepatic abscess and result in biliary bleeding and delayed liver rupture [18, 19]. Thus, we should carefully evaluate the severity of each case to perform ERC.

In our study, two patients had traumatic injuries of the central bile duct, both of which occurred at the hepatic bifurcation and were complex and completely separated; both cases were successfully treated with hepaticojejunostomy or hepaticojejunostomy with liver resection. If the central bile duct injury can be repaired by primary suture, ERC and internal stenting for biliary decompression should be considered an option [20]. However, detecting the degree of bile duct injuries with massive fluid collection or abdominal effusion close to the injury location, such as those in grade III-V injuries, on CT or MRCP preoperatively is difficult. Currently, there is no treatment algorithm for central bile duct injury available in the literature, and management of the injury must be individualized.

HAPs can occur even in grade II injuries if liver parenchymal injury is found deep in the liver parenchyma. Because our cases had a diameter of < 10 mm and were surrounded by the liver parenchyma within the intrahepatic parenchyma, we chose to treat conservatively. Kittaka et al. reported that post-traumatic pseudoaneurysm in solid organs, such as the liver, spleen, and kidney, may disappear spontaneously when the diameter is < 10 mm [21]. Even if the HAPs rupture, it might be possible that hemostasis could still be obtained by internal pressure. On the other hand, HAPs exposed on the damaged parenchymal surface of the liver in patients with grade III-V injuries should be treated with TAE regardless of their size, because once ruptured, they can bleed into the peritoneal cavity and can be fatal. The frequency of death from aneurysmal rupture after liver trauma is unknown; however, the reported mortality rate after visceral artery aneurysm rupture into the peritoneal cavity is relatively high at 35% [22]. Therefore, we performed emergent TAE in a grade III liver trauma patient, which had a diameter of only 4 mm.

All of our HAP cases were located on the peripheral side of the artery; however, treatment options are available for the central side. For patients with HAPs occurring in the common hepatic artery, coil embolization is performed, with the expectation that collateral circulation to the liver will develop from the gastroduodenal artery. For patients with HAPs occurring in the proper, right, or left hepatic artery, we have a plan to use a covered metallic stent normally used for coronary artery diseases, as it can maintain the hepatic blood flow.

One recent systematic review reported that hepatic necrosis is a common complication after TAE for the treatment of severe liver trauma, with a weighted mean rate of 15% [23]. A previous study also reported that hepatic necrosis tends to occur in severe liver trauma and that management of hepatic necrosis can be challenging [24]. As we avoided performing TAE in one case, if we recognize that interventional therapy is not needed (i.e., because the pseudoaneurysm is unchanging or it has a high risk of liver necrosis), we do not perform TAE. However, at present, there are no clear criteria for TAE for hepatic artery aneurysm and should be treated depending on the case.

This study has some limitations. This was a retrospective study performed in a single institution. Since the incidence of bile duct injury and HAPs are rare, the number of patients with delayed complications in this study was limited. However, this study is potentially important as it highlights the possibility and usefulness of conservative treatment for BL with peripheral bile duct injury. Future research is needed for expanding the study to multiple medical centers.

In conclusion, peripheral bile duct injury may be treated conservatively with intra-abdominal drainage, even in severe blunt liver trauma. However, injury of the central bile duct requires surgical treatment, such as liver resection with reconstruction of the injured bile ducts, once the patient’s condition is stable. Therefore, we believe that the management of post-traumatic BL depends on the location of bile duct injury. Assessment of the patient’s condition, including the location of the pseudoaneurysm and risk of liver necrosis, is important when performing TAE for HAPs. Our findings make a significant contribution to the current clinical management of delayed complications after severe blunt liver trauma.

## Data Availability

The datasets used and/or analyzed during the current study are available from the corresponding author on reasonable request.

## Abbreviations

AAST: American Association for the Surgery of Trauma
BL: Bile leakage
CT: Computed tomography
DCS: Damage control surgery
ERC: Endoscopic retrograde cholangiography
HAPs: Hepatic artery pseudoaneurysms
IABO: Intra-aortic balloon occlusion
ICU: Intensive care unit
ISS: Injury Severity Score
JATEC: Japan Advanced Trauma Evaluation and Care
POD: postoperative day
TAE: Transcatheter arterial embolization
